# Aerosol influenza transmission risk contours: A study of humid tropics versus winter temperate zone

**DOI:** 10.1186/1743-422X-7-98

**Published:** 2010-05-14

**Authors:** Brian P Hanley, Birthe Borup

**Affiliations:** 1 Butterfly Biosciences, PO Box 2363, Davis, CA 95616, USA; 2 BYK-Chemie GmbH, Abelstraße 45, 46483 Wesel, Germany

## Abstract

**Background:**

In recent years, much attention has been given to the spread of influenza around the world. With the continuing human outbreak of H5N1 beginning in 2003 and the H1N1 pandemic in 2009, focus on influenza and other respiratory viruses has been increased. It has been accepted for decades that international travel via jet aircraft is a major vector for global spread of influenza, and epidemiological differences between tropical and temperate regions observed. Thus we wanted to study how indoor environmental conditions (enclosed locations) in the tropics and winter temperate zones contribute to the aerosol spread of influenza by travelers. To this end, a survey consisting of 632 readings of temperature (T) versus relative humidity (RH) in 389 different enclosed locations air travelers are likely to visit in 8 tropical nations were compared to 102 such readings in 2 Australian cities, including ground transport, hotels, shops, offices and other publicly accessible locations, along with 586 time course readings from aircraft.

**Results:**

An influenza transmission risk contour map was developed for T versus RH. Empirical equations were created for estimating: 1. risk relative to temperature and RH, and 2. time parameterized influenza transmission risk. Using the transmission risk contours and equations, transmission risk for each country's locations was compared with influenza reports from the countries. Higher risk enclosed locations in the tropics included new automobile transport, luxury buses, luxury hotels, and bank branches. Most temperate locations were high risk.

**Conclusion:**

Environmental control is recommended for public health mitigation focused on higher risk enclosed locations. Public health can make use of the methods developed to track potential vulnerability to aerosol influenza. The methods presented can also be used in influenza modeling. Accounting for differential aerosol transmission using T and RH can potentially explain anomalies of influenza epidemiology in addition to seasonality in temperate climates.

## Background

The contrasting epidemiology of influenza in the tropics versus temperate regions has been discussed for many years, and it has been accepted for decades that jet aircraft are a major vector for global spread of influenza[1]. This study is an attempt to better understand aerosol influenza transmission for indoor locations by examining temperature and humidity indoors where jet travelers are likely to interact with locals and comparing humid tropical locations with temperate winter ones. In recent years, much attention has been given to the spread of influenza around the world, especially with the continuing H5N1 outbreaks since 2003 and the H1N1 pandemic in 2009. Extensive research has been conducted to understand the mechanism of transmission of influenza virus, including environmental conditions that favor transmission. Various aerosol studies have shown that micron range droplet particles from breathing, talking, coughing and sneezing bear influenza viruses, and that the aerosol route is an important contributor to infection[2,3]. The particles making up aerosol in normal exhalation are less than 1 micron in size; aerosol particles range from 0.1 micron to 5 micron[2,4], and these smallest particles are primary vectors of contagion[5,6].

Questions have been raised as to whether or not aerosol transmission of influenza occurs or is a significant contributor to its epidemiology, and whether vitamin D is a determining factor[7-10]. We believe that our study sheds helpful light on these matters by defining a framework that starts to formalize the effect of temperature and RH conditions on such transmission. We treat this more extensively in the discussion section.

We intend this study to be primarily targeted at public health planners and epidemic model developers. Interventions to successfully interrupt spread of influenza that have been studied in depth are quarantine, isolation, different types of masks, gloves, hygiene, and combinations of these[11]. Public health planners can use our results to consider making climate control adjustment recommendations, which can help control aerosol transmission. As well, modeling of epidemics in software depends on assumptions about where contagion is likely to occur. Some types of modeling today may take into account generalized types of mixing locations which are enclosed[12,13], as it is believed that most transmission (including aerosol) occurs indoors, with much attention put on social network[14,15]. We believe that such models can be improved by modeling of interior temperature and RH.

The authors developed a contour map of T versus RH based on literature from Lowen et al.[16-18] and others. In the studies of Lowen et al. guinea pigs were exposed to aerosol infection from another guinea pig for 7 days in an environmental cabinet maintaining temperature and relative humidity at varying levels. Thus, where we refer to a 25% risk of transmission, or a 25% contour, we mean that the risk of aerosol infection of one guinea pig over 7 days of continuous aerosol exposure to an infected guinea pig is roughly 25% (25%_G7_). We use this animal model as a baseline for estimation of differential risk to human populations. It is understood that temperature and humidity are not the only factors in aerosol transmission; however, we believe that they are primary factors along with dilution by air exchange and distribution by air currents[2,19]. In modern building systems, recirculation of indoor air for energy efficiency is also a likely factor. We collected data in 8 countries in the tropics and 2 Australian cities during winter (June-September 2009). Relative humidity and temperature readings were taken in public areas frequented by travelers (e.g. hotels, banks, malls, shops, taxis, buses, etc.) as well as during flights between nations. Observations were also recorded of behaviors that could augment the spread of influenza significantly. Interviews were conducted in major cities in the tropics and Australia to improve understanding of influenza transmission conditions.

In the process of our study, observations were made that suggest inexpensive measures that could be taken to minimize the spread of influenza in the tropics via aerosol, and these may also apply to temperate regions.

## Methods

### Instruments and readings

A model EP8706 digital psychrometer (Mannix Testing & Measurement, Lynbrook, New York) was used to take readings of temperature and humidity for most of the data. For a subset of the Singapore data, a Holmes analog humidity meter was used with a conventional analog dry bulb thermometer. These instruments were calibrated against each other, and the digital psychrometer was assumed to be the more accurate instrument. Correction was applied to the Holmes humidity data based on cross-calibration. Each reading is a one-time measurement; instruments came to equilibrium before a reading was recorded by hand with the time, date and location. Care was taken to ensure instrument temperature was at ambient, shielded from strong air currents and major infrared radiant sources to prevent condensation or improper evaporation from the probe.

### Locations surveyed

During the months of July through September 2009, major cities in the countries of Costa Rica, El Salvador, Nicaragua, Panama, Peru, Thailand, Singapore and New Guinea were surveyed as representative of the humid tropical belt. Australia was chosen as the location of cities experiencing temperate winter conditions and a significant spike in H1N1 influenza. The cities (which included nearby suburban areas) were: San Jose, Costa Rica; Lima, Peru; Panama City, Panama; San Salvador, El Salvador; Managua, Nicaragua; Bangkok, Thailand; Singapore; Port Moresby, Papua New Guinea; Sydney and Melbourne, Australia. Some surveillance of outlying areas was also performed.

Travel locations typical of choices made by tourists and business travelers was performed, as well as a survey of shops, offices, malls, high end hotels, and dining establishments in each city. These locations were chosen because in the modern world, epidemics are spread rapidly by aircraft[1] and thus by extension, the places visited by those who travel on aircraft (tourists and businesspeople) are the logical contact points with the population of those nations.

Environment in buses, tour cars, and taxicabs was also monitored, and, where feasible, time courses were conducted on transportation. These data could be improved on by a more comprehensive data collection conducted in more countries over the course of several years at a larger number of locations. However, the data is sufficient to be useful, and care was taken to make choices as consistent with tourist and business behavior typical of air travelers as possible and to only observe, never direct or interfere with environmental controls or human behavior.

### Contagion estimation contour development

**Figure 1:**
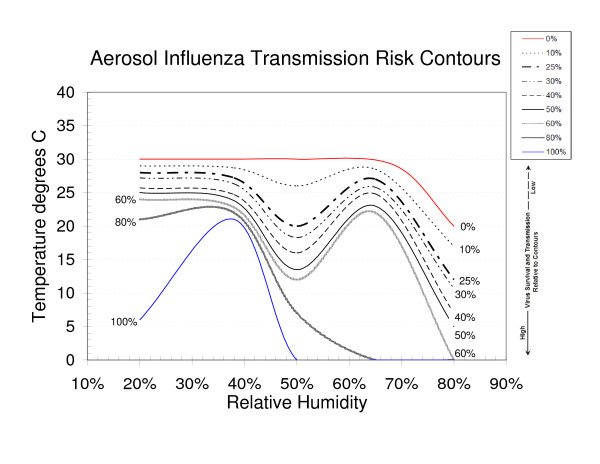
**Aerosol influenza transmission risk contours**. For the purposes of estimating risk of transmission of influenza by aerosol over 7 days, data points consisting of relative humidity and temperature in degrees C can be plotted on this contour map of the risk of transmission. (See Discussion, **Development of contagion contours**.) The risk of transmission over 7 days is the percentage value called out for each contour line. The 25%_G7 _transmission risk contour is emphasized (bold) as a boundary for risk of contagion. The 25%_G7 _contour was chosen based on calculating estimates of *R *(reproductive) values for various locations based on crude risk of aerosol transmission as derived from Equation 2, **Contagion probability estimate by time expressed in days**. (See also Discussion, **25%**_**G7 **_**transmission risk contour selection**.)

After review, aerosol transmission estimations were primarily drawn from Lowen et al. to interpolate contours of transmission of influenza in the humidity versus temperature phase space. A set of contours was generated that is believed to be mildly conservative (Figure 1 and Additional file 1 - **Contagion contour estimation details**). The emphasized 25% transmission line corresponds to a rough 25% transmission probability in the Lowen et al. guinea pig studies, which were done with continuous exposure for 7 days with animals housed near each other on a shelf (25%_G7_). This line was used as the cutoff when counting locations inside and outside of optimum aerosol transmission conditions. Similarly, other contours can be referred to with the G7 subscript to clarify what is being discussed, (e.g. 10%_G7_, 50%_G7_, 100%_G7_, etc.)

Equation 1 is a polynomial equation fitted for the 25%_G7 _contour using Maple 10[20] (Maplesoft, Waterloo, Ontario, Canada) and entered into Excel 2003 (Microsoft, Redmond, Washington). This equation was used to create the distance values for other figures. Due to the length and technicality, equation 1 is only shown in the additional files. (Additional file 1- **Contagion contour estimation details, *Equation 1: 25%*_***G7 ***_*risk contour temperature***.) A sample equation formatted for spreadsheet use (Additional file 2 - **Empirical 25% line equation in text format for use in Excel**) is supplied in text form along with the Maple 10 file used to generate it (Additional file 3 - **Maple workbook for 25% line equation**). Note the equation fit is meaningless below 20% RH or above 80% RH.

### Morbidity and mortality data

We recognize that accurate influenza morbidity and mortality data are notoriously difficult to acquire[21,22]. Mortality and morbidity rates based on current surveillance data were collected both from official reports (WHO PAHO/SEARO, Departments of Public Health) and directly from responsible public health personnel in interviews. Care was taken to try to minimize artifact differences due to surveillance deficits. Alternate sources such as Flutracker[23] were consulted to vet surveillance data and we believe our data is as accurate as obtainable for the period.

### Crude risk calculation

**Figure 2:**
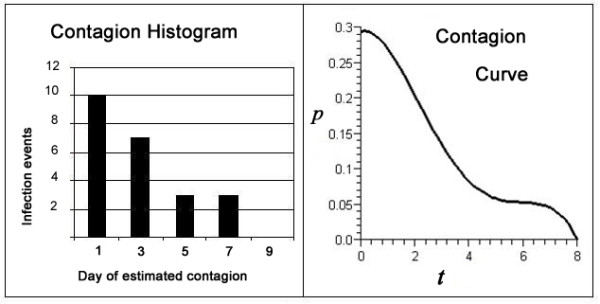
The contagion curve is the plot of an equation fitted to the contagion histogram  using Maple 10. (See equation 2, **Contagion probability estimate by time expressed in  days** and Additional file 1 - **Contagion contour estimation details**, Equation 2.) The  zero for the contagion curve is adjusted to reflect the probable day of contagion, rather  than when viral loads appear. The contagion equation provides a conservative  continuous probability of the infection of one individual exposed to infection by aerosol  versus time expressed in days.

Crude risk derivation is based on a histogram of the estimated time of contagion for the animals in Lowen et al., as presented in Figure 2. Calculated crude risk estimates are given in tables where time in location estimation allows it.

A polynomial equation (equation 2, Additional file 1- **Contagion contour estimation details**) was fitted to the histogram data using Maple 10[20] to estimate the risk of start of infection over the days in which secondary infections are estimated to have occurred. This 4^th ^degree equation from day 1 to 9 was selected to fit observations and knowledge that virus shedding from inoculated animals is expected to end by day 8. To express probability of infection, the histogram values were represented as fractions of the total number of infections, and the equation fitted so the area under the curve is equal to 1 to 3 decimal places. Tables 1 and 2 contain estimates generated from this equation. (See also: Additional file 4 - **Maple workbook for empirical contagion probability equation**, and Additional file 5 - **Empirical contagion probability integral in text format for use in Excel**.)

**Table 1:** Crude risk of contagion in vehicles or buildings

	25%_G7 _transmission risk contour line		40%_G7 _transmission risk contour line		60%_G7 _transmission risk contour line	
	Per passenger or patron	Per vehicle or building	Per passenger or patron	Per vehicle or building	Per passenger or patron	Per vehicle or building
Luxury bus 4 hr	*1/81*	0.6	*1/51*	1	*1/34*	2
Luxury bus 8 hr	*1/41*	1	*1/25*	2	*1/17*	3
Luxury bus 12 hr	*1/27*	2	*1/17*	3	*1/11*	4
Taxi 20 min	*1/989*	1/495	*1/618*	1/309	*1/412*	1/206
Taxi 45 min	*1/435*	1/218	*1/272*	1/136	*1/181*	1/91
Tour car 2 hr	*1/163*	1/82	*1/102*	1/51	*1/68*	1/34
Tour car 5 hr	*1/65*	1/33	*1/41*	1/20	*1/27*	1/14
Bank branch 10 min	*1/1959*	1/109	*1/1224*	1/68	*1/816*	1/45

Crude risk of contagion for individuals in vehicle or building locations assuming presence of virus shedding. Estimates are calculated from equation 2 expressed as whole number fractions for selected transmission probability contours. For example, the per passenger risk on a 4 hour luxury bus ride is 0.0123, or 1/81. Since buses are assumed to have 50 passengers, for conditions at the 25%_G7 _risk contour line, 50/81 = 0.6. So for every two bus rides of 4 hours under these conditions, approximately 1 aerosol infection would be expected. Similarly, in 10 minutes in a bank branch at the 25%_G7 _risk contour, assuming 18 people in the branch, there is a roughly 1 in 1959 chance of infection in any 10 minute period. Assuming 300 patrons per day, there would be 1 infection per branch during a one week infectious cycle under standard conditions.

For quick, rough estimates in a public health setting, estimates such as these can be made by use of the table. These kinds of estimates were used to decide which was the reasonable aerosol transmission risk contour to use to demarcate the low risk boundary of the figure 1 graph. (See Discussion, **25%**_**G7 **_**transmission risk contour selection**.) It should be noted that these numbers are useful as guidance rather than being completely predictive since other factors such as ventilation diluting virus aerosol, rate of exhalation of aerosol virus, direction of airflow, and wake effects will have major effects on actual infection numbers.

Equation 2: Contagion probability estimate by time expressed in days

*Where **t** =time expressed in days*, ***p ****is crude risk probability assuming 100% 7 day contagion (100%*_*G7*_) ***C(T, RH) ****is the estimated risk for a field reading from the contour map and ****p***_***c ***_= *probability of contagion. Result ****p ****when multiplied by contagion contour percentage at a temperature and RH provides the time scaled risk of infection in a location (****p***_***c***_). See Additional file 1- **Contagion contour estimation details*** for more discussion*.

## Results

**Figure 3:**
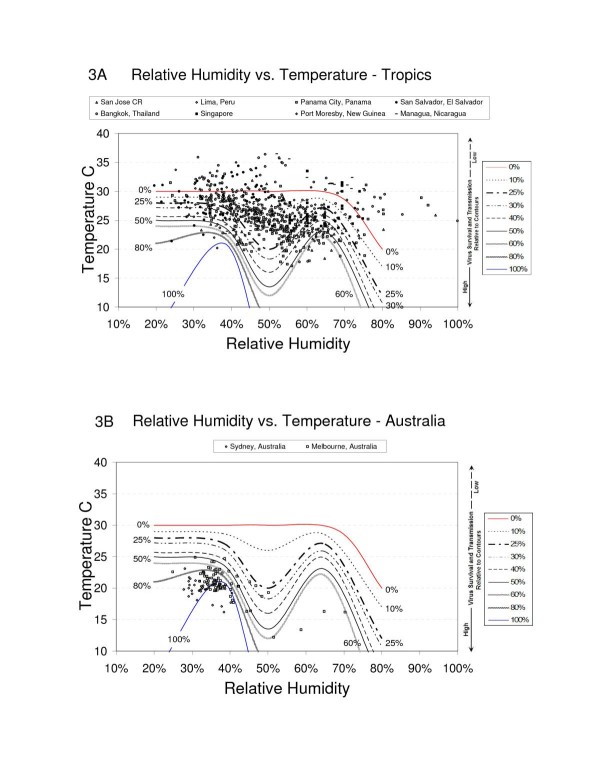
**Relative humidity versus temperature for all data**. In these scatterplots the difference between tropical nations (3A, N = 766) and Australia's temperate cities in southern hemisphere winter (3B, N = 110) can be seen on a contour map of risk of transmission by aerosol. The contour lines are based on aerosol transmission time course experiments over 7 days by Lowen et al. together with support from other literature. Thus, the 25%_G7 _transmission line indicates that over 7 days under similar controlled conditions 25% of hosts exposed by aerosol would become ill. (See equation 2, **Contagion probability estimate by time expressed in days** for how to calculate risk based on time in location.) For general purposes of evaluating risk, the distance of points below the chosen transmission line can be visually evaluated by inspection. In these graphs, datapoints consist of temperature and RH for: Bank Branch, Club, Casino, Church, College, Dining, Dwellings, Elevator, Entertainment, Gym, Hospital, Hotel, Mall, Office, Pharmacy, Public site, Retail, Terminal, and Transport, for all nations. To see cumulative distribution data showing distance from the 25%_G7 _transmission risk contour line for these data, refer to Figure 4.

**Figure 4:**
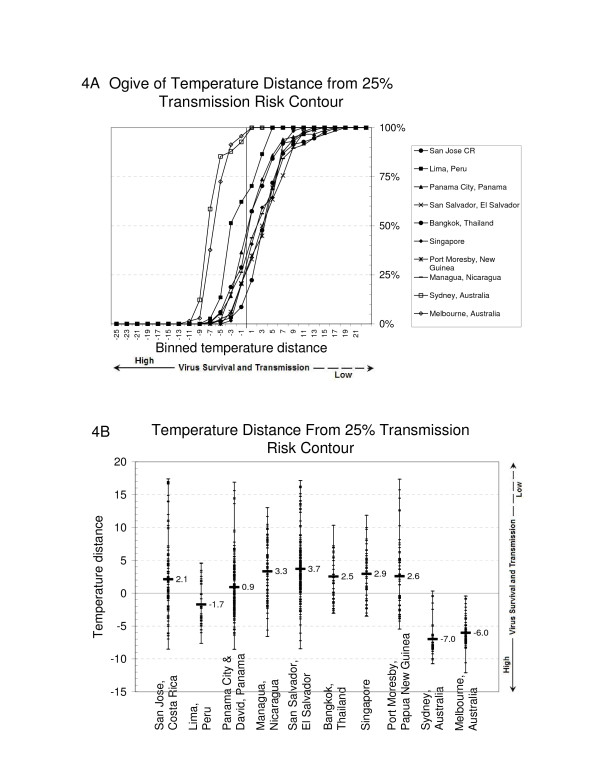
**Temperature distance from 25%_G7 _transmission risk contour**. The 4A ogive (cumulative distribution curve for the histogram) for each nation was plotted with a vertical line at the zero point. For Figure 4A bins of 2 degrees C width are used to categorize temperature distances from the 25%_G7 _transmission risk contour. Figure 4B presents the same temperature distance data in a linear density plot for each nation, with a tick mark for each temperature distance, and the arithmetic mean. The temperature distances were calculated for each point shown in Figure 3 using a Maple generated 25%_G7 _transmission risk contour line equation. (See equation 1, and Additional file 1- **Contagion contour estimation details** for equation and details of use.) For both figures, the zero point of these graphs is the 25%_G7 _transmission risk contour line.

**Figure 5:**
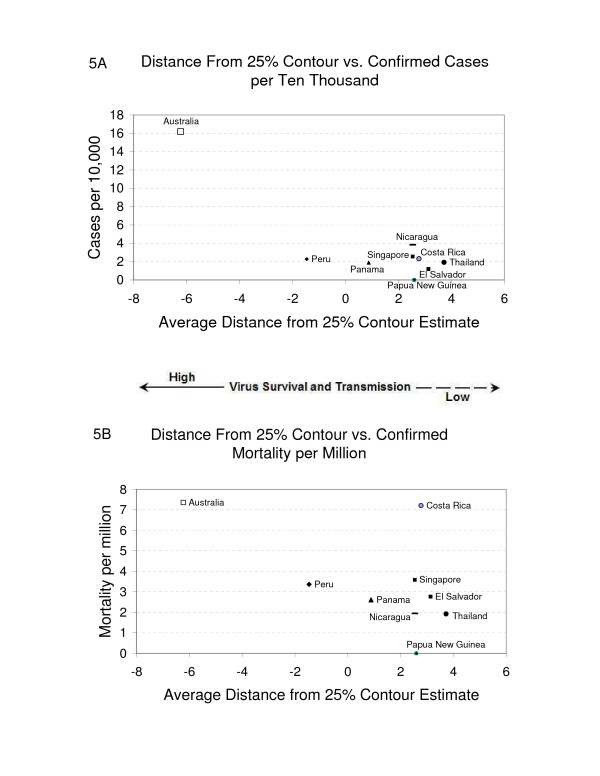
**Case and mortality reports versus average distance from 25%_G7 _transmission risk contour**. These figures show the relationship between the average temperature distances as shown in Figure 4, and influenza case reports per 10,000 (5A) or influenza case mortality per million (5B). Case and mortality figures are up to September, 2009.

The aerosol transmission contours are presented in Figure 3 with all data plotted by nation. Temperature dimension distances from the 25%_G7 _transmission line for all data points produced the cumulative histogram of temperature distances ogive/density charts of Figure 4. This shows the relationship of readings to the 25%_G7 _risk contour for the entire dataset. Figure 5 shows correlation of average distance from the 25%_G7 _contour to cases of influenza. We will now examine in more detail different types of locations surveyed.

### Buses and taxis

**Figure 6:**
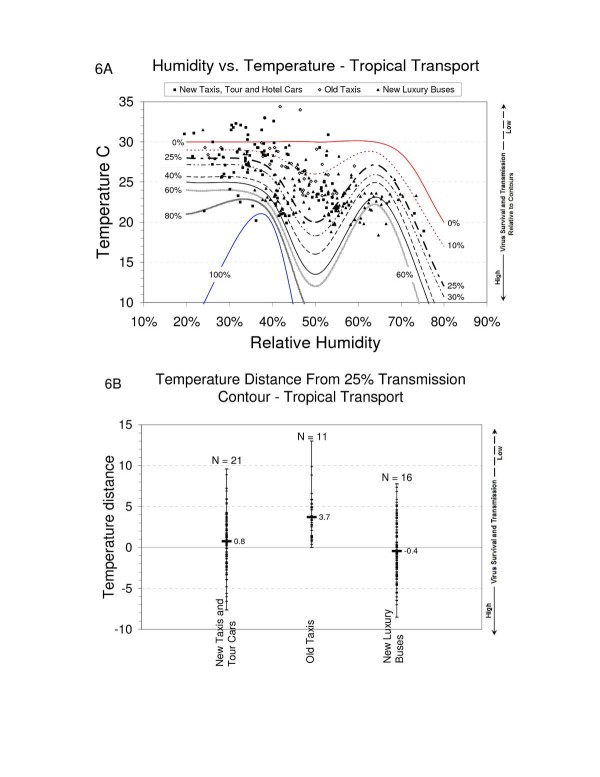
**Closed, Air Conditioned Tropical Ground Transport**. The scatter plot (6A) and linear density plot (6B) show all data (multiple time course readings per trip) for motor vehicles categorized by old and new. The vehicle data shown above is for vehicles with closed windows and air conditioning. All old taxi, tour and hotel car readings show low risk (above the 25%_G7 _transmission risk line). Most readings for new cars in this low risk region are due to higher temperature and low humidity, presumably because automotive engineers are using evaporative cooling from the skin of passengers in dehumidified air to lower perceived temperature.

Luxury buses are used for long-distance trips of 4-12 hours, have air conditioning (AC) and windows operable only in emergency. A not uncommon complaint is that these buses are too cold. They are commonly used by traveling visitors, and also by middle class inhabitants of the region desiring more comfort in their travel. Luxury bus trips (N = 16) fell into the region of concern below the 25%_G7 _transmission risk contour (Figure 6), and passengers shedding virus are a probable source of aerosol. Additionally, vendors often appear at stops selling merchandise. Some vendors get on at one stop and travel with the bus for 45 minutes or so, selling products and entertaining the passengers. Others get on the bus at stops and spend shorter periods of time on the order of 10 minutes, getting off one stop later. Some get on and off at the same stop in 2-4 minutes. Total estimated vendor time inside buses is on the order of 3-5 hours per day over as many as 20 buses, each bus containing potential influenza aerosol, raising their chances of both infection and transmission. Wake effects such as those proposed to explain SARS transmission in a modern jet aircraft[24] are generated by them, and they lean in close to many passengers. Additionally, they have physical contact through money and product exchange, which is usually food.

Inexpensive buses used by locals have no AC, using open windows for ventilation. Readings confirmed that their temperatures and RH are equal to or higher than ambient temperatures (data not shown). Vendors serving these inexpensive buses were fewer, and were only observed to board briefly at stops, departing the bus within 2-3 minutes, or to make sales directly through windows.

**Figure 7:**
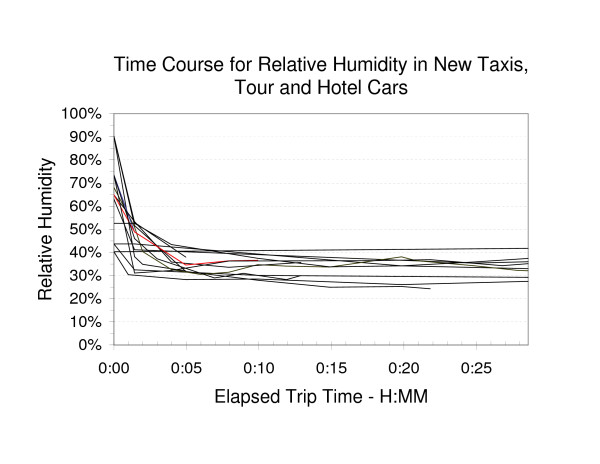
**Relative humidity time course for new tropical automobiles**. In this figure are shown automobile trips of 5 minutes or more in closed vehicles with air conditioning. It can be seen that within 5 minutes, relative humidity is lowered to between 45% and 25%. Time 0:00 is street ambient temperature and the first interior reading is at or after 1 minute. The humidity in a new automobile was significantly lower than the street humidity at the time of the first reading after the door was closed. That these vehicles recirculated their air would be expected to contribute to contagion. N = 15.

Trips in high end taxicabs and new minivan shuttles (N = 21) also showed good transmission conditions (Figure 6). Trips in old taxis (N = 11), even if closed and air-conditioned, and those not using recirculation, stayed out of the high risk region. The ability of a new automobile to rapidly lower humidity with the windows closed on recirculation setting to between 45% and 25% RH within 5 minutes or less is remarkable (Figure 7). New high end taxis and hotel cars were commonly occupied by travelers for 20-45 minutes (to/from airports, to/from holiday or business meetings).

**Figure 8:**
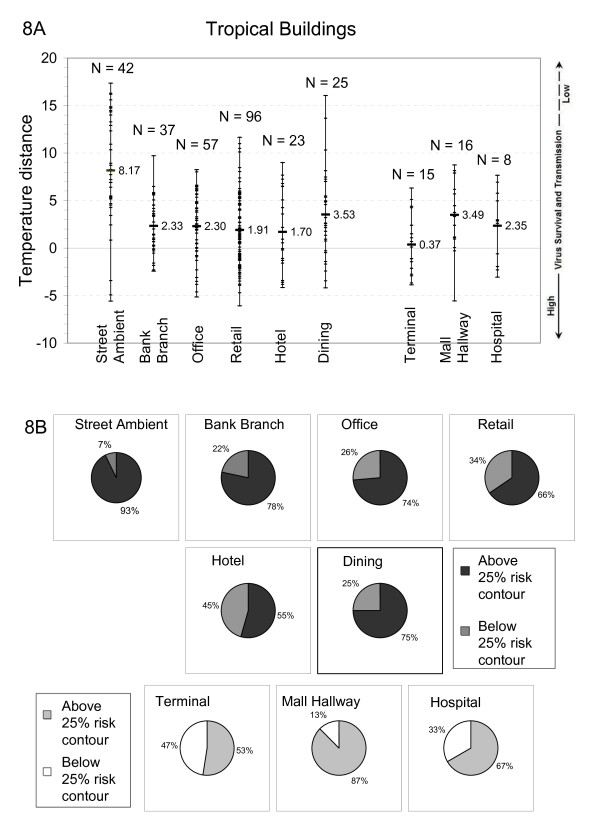
**Tropical buildings distance (in temperature) from 25%_G7 _transmission risk contour**. In this figure, outdoor conditions (street ambient values) contrast with conditions inside of buildings. 20% or more of the bank branch, office, retail, hotel and dining locations displayed good aerosol transmission conditions (i.e. below the 25%_G7 _transmission line) with sample size > 20. Temperature and RH of terminals (for aircraft and buses), hallways in shopping malls, and hospitals, was considered indicative and thus shown, though having smaller sample sizes than desired. Although there were only 8 different hospitals surveyed in tropical nations, hospital data was included because hospitals receive ill patients, and many of these nations have only one major facility. These data suggest that hospitals have a relatively high investment in HVAC in the tropics, on the order of retail and high end hotels, presumably for comfort of patients. Black and dark gray pie charts are shown for datasets with N greater than 20. Datasets with less than 20 measurements in the sample are shown with light gray and white to emphasize the difference in sample size.

### Non-residential buildings

Of high end tropical hotels studied (N = 22) approximately 50% had good conditions for aerosol transmission in common areas. However, all tropical hotels having good conditions were in the high RH region near 65% (data not shown).

Tropical locations to find good conditions for aerosol influenza transmission to the general public were bank branches, dining facilities, retail shops and offices (Figure 8). The overall impression was that business locations in the tropics needing to appear high-status set their AC systems to generate low humidity and temperature.

In the temperate Australian winter, 98% of buildings (N = 100) showed good aerosol transmission conditions (Figure 3B). Thus, no breakdown by type of facility is presented.

### Dwellings

Dwellings were not surveyed in all nations and the sample was low; however, where surveyed, estimates were made of how typical RH and temperature were for a dwelling class. The majority of tropical apartment buildings (N = 10, data not shown) had open-air common areas and showed poor aerosol conditions. Surprisingly, temperature and RH conditions in dwellings surveyed were not optimum for transmission in Australia, although the sample was insufficiently large.

**Figure 9:**
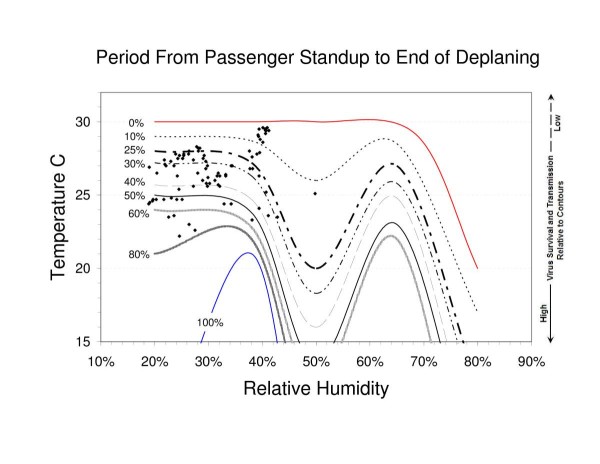
**Deplane period**. This figure shows average temperature and relative humidity from the time the first passenger stands up in each aircraft until completion of deplaning for all datapoints in the time courses. Aircraft N = 15.

### Airplane flights

**Figure 10:**
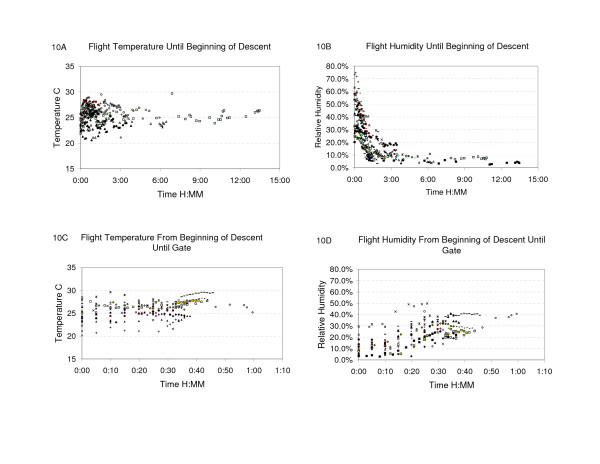
**Time course plots of plane flight temperature and relative humidity**. 10A and 10B show temperature and RH from the time of leaving the gate until the aircraft begins its descent to land. 10C and 10D show temperature and RH from the time of starting to descend for landing until passengers leave the plane (where zero is the start of descent toward landing). Influenza transmission risk evaluation is not attempted due to lack of transmission data in literature for most of the humidity levels, HEPA filtration and other factors. Graphs are provided for informational interest. (See Discussion, **Aircraft data interpretation**.) Aircraft N = 15.

Using our transmission contours, conditions for influenza transmission exist during deplaning (Figure 9) for intervals of 7 minutes or less from the time passengers stood up until aircraft cleared (mean 3 min 55 sec, N = 12). During this time, ventilation was often turned to a low setting or off. The authors believe risk is probably low on a per passenger basis, because the period is limited to the short time in which passengers file out of the airplane and other factors may override T and RH. Aircraft data (Figure 10) is otherwise presented without risk interpretation (see discussion).

**Table 2:** Risk of transmission during deplaning

	25%_G7 _transmission risk contour line		40%_G7 _transmission risk contour line		60%_G7 _transmission risk contour line	
	Per passenger	Per aircraft	Per passenger	Per aircraft	Per passenger	Per aircraft
Mean airplane deplane period 5 min						
Range: 2 to 13 min	1/3917	1/20	1/2448	1/12	1/1632	1/8

Shows risk of contagion during the deplaning period of a flight, assuming an infected passenger shedding virus, assuming 200 passengers per aircraft. During deplaning, 22 of 87 time intervals of 2 minutes were above the 25%_G7 _transmission risk contour, and 5 of 87 intervals were below the 60%_G7 _contour. Deplaning mean contour was approximately 40%_G7_. Moser[34] has a time period of 4.5 hours in an unventilated enclosed aircraft on March 14, 1977 in Homer, Alaska when the outdoor high was 0°C (outdoor RH ranged from 67% to 96%) while the aircraft sat on the runway with the ventilation off. In that incident, 39 of 54 passengers (72%) were reported ill from an index case. Using Equation 2 with 100%_G7 _transmission risk, approximately 3 new infections would be expected, or roughly 10% of what was seen, which is a reasonable correlation given an enclosed space without ventilation and uncertainties about conditions. As others have noted, this confirms that factors such as ventilation have a strong impact on aerosol transmission[50]. Aerosol infectious virus load per unit of air would be expected to vary based on rate of input, rate of deactivation, and degree of dilution by ventilation. We think it is reasonable to include a multiplier on estimates where air exchange is poor and that 10× is a reasonable upper bound.

## Discussion

### Development of contagion contours

Literature shows opposing conditions for transmission of viruses in general; low relative humidity (RH) and high RH[5,25,26] with temperature a secondary factor. Theory predicts osmotic forces should affect enveloped viruses such as influenza, while icosahedral viruses (e.g. polio, norovirus) would not be so sensitive for structural reasons. Enveloped viruses generally have highest infective stability at RH somewhat below 50%[5], and non-enveloped icosahedral viruses usually show greatest infective stability in aerosol in high humidity conditions[25,26].

Data of Lowen et al.[16,17] at 20°C show optimal transmission of influenza by aerosol at a first RH range from 20-40%, and at a second from 60-70%. Lower temperatures improve transmission, with temperatures above 30°C reducing transmission to zero. These data correlate with other *in-vitro *studies[5,25-27].

Influenza is an enveloped virus. Enveloped viruses bud from the cell membrane, so the virus envelope is host cell (or golgi) membrane acquired in the budding process. Inside the envelope is RNA, a few enzymes and proteins, along with cell cytosol at physiological salt concentration. This matters because if one puts a cell in an environment containing lower salt concentration than in cytosol, the cell membrane acts as an osmosis membrane and eventually ruptures[28]. Enveloped viruses will have the same issue, although the smaller diameter should give greater stability to rupture per equation 3.

Equation 3:

*Where F *= *membrane tensile force, P *= *pressure, r *= *radius*

Infectious droplets from the lungs start out with physiological levels of salts. These salts could cause rupture of virion envelopes as droplets collect distilled water from humid air. Schaffer et al[27] studied stability of enveloped viruses from different cell lines (viz. kidney, chick embryo) and these cell lines buffer osmotic pressures at different rates[28]. Those results indicate that cells which are better at buffering themselves to osmotic pressure produce enveloped viruses that survive longer at higher RH. We are not aware of any direct study of osmotic destruction of enveloped virions, although it makes considerable sense.

At lower RH, enveloped viruses are quite stable and infectious; at high RH they are not. One possibility is the above-mentioned osmotic pressure issue. Another is the theory that droplet particles settle more quickly as they take on water[17] under high RH, which fits Stokes' law[2]. In addition, enlargement of the particle as a condensation nucleus will cause it not to penetrate as far into lungs as a result[2]. However, none of these hypotheses explain the viability trough at 50% RH nor the secondary peak at 65% RH, although the rapid decline toward 80% RH does fit. The primary variable between *in-vitro *studies and Lowen et al. appears to be differences between the synthetic droplet media of the *in-vitro *studies[25,27] and the natural droplets from exhalation, which are likely to be glycoproteins, salts and other components of mucus[29].

A study by Harper[25] examined, *in-vitro*, survival of 4 cultured viruses in aerosol, at temperatures ranging from 21°C to 24°C. To the degree his results differ from Lowen in the 50% + humidity range, they might be explained by his higher temperature. If so, that would change the contours of influenza transmission risk (Figure 1) somewhat, although the current transmission risk contours would remain conservative. Alternatively, this difference may be from the droplet fluid carrying virus used by Harper, as mentioned above.

There is an argument that influenza strains might vary in stability from mutations sufficiently to affect the contours of transmission as taken from Lowen. However, evolutionary argument supports virus stability in aerosol as strongly conserved, since in humans, viruses with lesser aerosol stability will not propagate as well as those with greater stability (unless aerosol stability is compensated for by some other propagation enhancement), and viruses with optimum stability will be selected for during host to host transmission[30,31]. Thus, literature results from human influenza virus strains would be expected to be from virus near the practical limit of aerosol stability. Further, osmotic pressure generates tensile force on the envelope, which will exhibit resistance to osmotic pressure not exceeding the weakest envelope bilayer hydrogen bonds.

Based on the considerations above, contours were generated based on linear interpolation of Lowen et al.[16,17] cross-validated with others[5,25,27,32]. These contours apply to RH conditions from 20% to 80%, although it is likely that contours above 80% RH have lower transmission risk than at 80%. Both the region from 0% to 20% RH and that above 80% RH are less clear and need investigation. The justification for using these risk contours in larger scale environments is based on data from studies that show long term persistence (hours) of viable aerosol virus[25,27].

### Statistical validity of the contour graph

As presented by Lowen et al.[16,17] in studies of aerosol transmission of influenza over 7 days, there are three temperature groups, 5°C, 20°C and 30°C at varying RH. For the 5°C temperature there are four RH categories, 35%, 50%, 65% and 80%. At 20°C and 30°C there is an additional fifth at 20% RH. At 30°C there is no transmission. At 5°C transmission varies from 100% to 50% and at 20°C from 100% to 0%. Thus, where statistical power is in question is between 5°C and 20°C. As discussed[17], the difference in transmissibility between 5°C and 20°C at 50% and 80% humidity is significant (*p *< 0.05). This leaves the 65% relative humidity results at 20°C to be examined.

To further evaluate the Lowen data, we considered it in the context of Harper[25] and Schaffer[27] data on time course viability of influenza virions at differing temperature and humidity, because it is axiomatic that the longer virions can remain viable in aerosol, the more likely they are to cause infection by this route. Harper shows support for the transmission decline of Lowen, as viability declines when RH increases toward 50%. Schaffer data for one hour survival at 21°C (see figure two of Schaffer et al.) also shows a viability trough at 50% RH rising at humidity above 50% followed by a decline[27]. These features of Harper and Schaffer further support the Lowen 20°C data for 50% RH, which was already of sufficient statistical significance. Additionally, Schaffer supports the 65% RH increase in transmission called out as statistically of insufficient power by Lowen et al. A further argument in favor of the 65% RH increase in transmission is care to present conservative contagion contours where there is a question; thus we retained the feature showing a rise in contagion at 65% RH.

Consequently, although *p *values for Lowen et al. alone are insufficient for acceptance of the 65% RH rise in contagion, taking alternative data sources and conservatism into account, we retained the 65% RH feature. We understand that the details of the type of contour map we present may change with larger datasets between 5°C and 30°C and we strongly encourage performance of larger experiments with multiple strains of influenza and other respiratory viruses. It would be highly desirable to have a larger dataset on the order of 30 animals or more at each temperature and RH setting and more temperature and RH values.

### 25%_G7 _transmission risk contour selection

For visual inspection purposes the 25%_G7 _transmission estimate contour is emphasized and became the reference using the following rationale.

Since Lloyd-Smith et al. reported the SARS epidemic was primarily propagated by superspreaders infecting 4 or more people[33], we chose conditions that should limit spreading to infect 1 or 2 on average.

The guinea pig experiments of Lowen were performed for 7 days. In most public places such as banks and hotel lobbies with good conditions for transmission of influenza, the time people spend is on the order of 10 minutes. This corresponds by integration of equation 2 to a crude risk of 1/1959 for any one entry and exit at the 25%_G7 _contour. Thus, assuming 300 patrons per day yields approximately 1 case every 6 days for a usual branch, assuming an infected individual is shedding virus continuously. Risk on a bus ride of 8 hours at the 25%_G7 _contour yields a crude risk of 1/41, which roughly corresponds to 1 new infection per 8 hour bus ride assuming continuous virus shedding. Assuming an influenza case of 7 days virus shedding duration, we thought it improbable most locals would take more than two 8 hour luxury bus rides with 50 passengers per bus in that time span (vendors excluded) for a total of 2 new infections. (Table 1)

These crude risks represent the rationale of our risk cutoff in enclosed spaces. However, we do not think one contour cutoff is always appropriate.

### Aircraft data interpretation

A considerable amount of data was collected for aircraft, however, transmission risk on aircraft is complex. First, the influenza contagion space relative to temperature and RH on aircraft is mostly unknown since studies have not been done below 20% RH, and large portions of flights can occur with RH in the 3% to 15% range (Figure 10). How such extremely low RH affects transmission is unknown. Second, although influenza was communicated well to aircraft passengers circa 1979 during a ground delay (38 of 54 passengers in 4.5 hours, 1 index case) given lack of air circulation[34], HEPA filtration of recirculated cabin air on most aircraft today mitigates this hazard, together with outside air exchange in flight. Literature raises questions about efficacy of HEPA filters on aircraft[35]; however, the careful epidemiology of SARS on an aircraft[24] suggests HEPA filters and air exchange were fairly effective on that aircraft because of the apparent wake pattern of infection. That correlates with modeling of wake particles carried behind persons moving along the aisle[36]. SARS, like influenza, is an enveloped RNA virus of the same size, which likely has similar filtration characteristics. Third, on some aircraft, ozone is negligible due to catalytic units[37]. Ozone would be expected to deactivate virions significantly[38], but the extent this occurs at ozone levels of aircraft lacking catalytic units is unknown. One must also weigh ozone causing a possible increase in host susceptibility and worse course of disease[39], another unknown. Consequently, we believe risk, per our study criteria, to be low on aircraft outside deplaning, but worth continued attention.

### Alternative views on aerosol transmission of influenza

Examining literature questioning whether influenza is transmitted by aerosol, it appears a major question is the distance at which aerosol transmission of influenza occurs, and we contend that such transmission varies greatly with conditions. There is also a proposal that vitamin D hormone is a regulator of seasonal influenza incidence in the context of questions raised about influenza's fundamental epidemiology[10].

#### Aerosol question - Han et al. example

In the example of Han et al.[9] the researchers state that no aerosol transmission occurred because interview data indicated that the 9 infected parties out of 31 tour group members all either talked to or were coughed on directly by the index case over a 3 day period. (Aircraft infections in Han are left aside for the reasons discussed above.) Noting that in a warm, humid environment contact transmission would be the expected primary mode, a guess can be hazarded in light of the current study.

What the temperature, relative humidity or ventilation characteristics were for the indoor environments of this tour group are not known with precision. A best guess of aerosol expectation based on bus data for comparable outdoor conditions in the current study derived a low of 1 infection (7 hours, 40%_G7 _risk) and a high of roughly 5 - 6 infections (7 hours, 100%_G7 _risk, poor ventilation roughly doubling infections). (Better estimates would depend on actual T and RH, ventilation and other factors.) Leaving aside the reliability of interviews, the droplet (non-aerosol per Brankston et al.[8]) doses administered by talking and coughing (presumably at close range) will drop off quickly with distance due to conditions and dilution. And depending on RH, those droplets may evaporate into aerosol.

A few aerosol infections out of the 9 could easily be missed by the Han methodology due to overlap (i.e. a person who talked to the index case could be infected by aerosol). Whether there is significance to there being 2 to 9 times the number of infections guessed depends on more details than are available. Given the caveats, contact could account for the majority of cases in this example as could aerosol.

#### Aerosol question - Branktson et al. and Lemieux et al

The concerns of Lemieux et al.[7] and Brankston et al.[8] as to whether aerosol influenza infection is significant hinge on a number of matters. Primary among these is what aerosol transmission means, as defined in the differentiation of droplets ≥ 5 μm and aerosol ≤ 5 μm. We find Tellier's response to Lemieux et al. compelling[40]. To it, we add several notes.

We believe that influenza is transmitted both by contact and by aerosol. The conversion of droplets ≥ 5 μm to smaller size varies based on humidity and we reiterate Tellier's point that studies have shown viable virus after hours suspended in air[25,27]. Tropical temperature and humidity does not block transmission by contact, but does block transmission by aerosol[17]. Since it has long been observed that influenza epidemics are muted in the tropics, and they die out in temperate summers, this argues that aerosol transmission is necessary to sustain a rise in *R*_*0 *_above 1 in large populations.

#### Vitamin D hypothesis

A number of studies have presented data showing an inverse correlation between serum 25-hydroxyvitamin D (25-hD) levels and upper respiratory tract infection (URTI) and questions raised about influenza epidemiology[10,41-43]. 25-hD level has been proposed as the seasonal factor for influenza epidemics by correlating 25-hD level inversely with influenza season. That these groups maintained a differential year round is evidence in its favor as a factor and 25-hD has been shown to positively modulate mucosal immunity[44].

Notably though, summer URTI incidence is roughly half of winter for all groups; also, the least protected group (10 ng/ml of 25-hD) in summer had approximately 25% lower URTI incidence than the protected group (30 ng/ml of 25-hD) had in winter. Additionally, influenza spikes can occur in October and November when 25-hD levels are reported relatively high[45]. Thus, 25-hD is a probable influence but whether it is sufficient in itself to explain seasonality is at most an open question. Vitamin A (present in cod liver oil with 25-hD for some studies) may also be a significant factor since vitamin A shows a strong influence on measles[46], which is another enveloped virus spread by aerosol. Such factors need continued study in order to include them properly in our understanding and epidemiological models.

The present study presents a view of influenza epidemiology that needs to be carefully considered, as it can potentially explain certain apparent anomalies of influenza transmission such as aspects of superspreading and unexpectedly low secondary attack rates[10].

## Conclusions

Given the results of this survey and analysis of literature data, we recommend the following to help mitigate the spread of influenza in the broader context of recommending that influenza epidemiological studies try to account for and report temperature and RH data of indoor enclosed locations to the extent this is practical.

### Provide inexpensive tools to monitor environment

Digital psychrometers cost on the order of $100 to $160 in single quantities (larger purchases may be possible at lower cost). Based on visualizing the data collected, steps can be taken to attempt to either move higher risk environments in the direction of lower aerosol transmission risk, or else direct the use of other measures in those environments. A chart (Figure 1) should be inexpensive to distribute.

### Educate luxury bus, taxicab, and hotel car operators

Luxury buses deserve special attention, as they cross borders and travelers spend upwards of 4 hours in the environment. The close quarters of these vehicles' recirculating air are a good opportunity for aerosol transmission of influenza (and other respiratory diseases).

Vendors to luxury buses represent the potential to be superspreaders of influenza, visiting many buses, speaking and moving systematically through the bus for periods of time per bus from 2 to 45 minutes, spending totals of hours per day in buses in multiple visits. Vendors also have direct contacts with passengers, increasing their chances of acquiring and then transmitting infection. Superspreaders were important for the SARS epidemic, showing an unexpected distribution of infectors[33] and probably are for influenza. Bus companies distributing masks and hand sanitizer to vendors to protect them may yield benefits.

### Focus on major hotels, shops, offices, dining, malls and bank branches

Public health can educate maintenance staff of luxury hotels, newer malls (small and large), offices, dining establishments, banks, and colleges about caring for their environmental settings during a flu season. For those locations that have a need to portray an image of higher status, and hence comfort, how to balance that is a question for HVAC engineers.

### Influenza transmission on aircraft is probably fairly low

Based on our examination, we think that influenza transmission on aircraft is probably a not a serious risk most of the time, as discussed above, although the passenger numbers are quite large. Most of the risk appears to be off the aircraft, although wake effects can be troublesome, and T and RH may regulate whether wake effects can occur. Lacking viability data for the humidity range common on aircraft, how that works is clearly an open question. However, since the period starting when passengers stand up after landing to emptying the aircraft does fall within our parameters and is quite short, it may be an insignificant cost for airlines to flush cabin air from the end of the runway after landing until passengers leave the aircraft to further lower transmission risk on aircraft.

### Public health relative to other disease and temperature vs. RH

Many viruses and bacteria will display viability conditions opposite to influenza. Endemic disease threat such as *M. tuberculosis *should be weighed since TB is correlated with tropical climates[47], suggesting its aerosol transmission is optimum in high RH and warm temperature. TB is a hardy organism that forms culturable aerosol from coughing[48] but the aerobiology of transmission is not well explored[47]. Guinea pig model TB transmission studies in parallel with influenza exploring variations of temperature and RH relative to HEPA filtration and ultraviolet light as recommended by Nardell and Piessens[47] would be desirable. The TB concern indicates that in TB endemic regions humidity lower than 60% should be targeted on the transmission contour map (Figure 1). There are also commonalities between other measures that can minimize influenza aerosol contagion and measures against TB and other microbial aerosol (such as UV irradiation of upper air [49]).

### Summary

Climate control for enclosed spaces should be added to public health to control influenza epidemics. The range between 20% and 80% RH covers most human habitation outside of aircraft, and the region above 80% RH appears to be a low transmission risk, although both these regions should be explored. In the tropics, getting an indoor facility out of the region of highest risk should be simple and low or no cost. In temperate regions, controlling AC to stay out of the optimum transmission region may be more challenging. At a minimum, the low or no cost step of changing climate control parameters should not raise the *R*_*0 *_(reproductive number) of an influenza epidemic and will lower it considerably if seasonal influenza transmission is any guide. The authors hope for further refinement; however, this is an inexpensive starting point with highly probable benefits, which should be a net savings for nations. For those who perform epidemiological studies, analyzing data in light of temperature and relative humidity will help our understanding of influenza epidemiology.

## List of abbreviations

25%_G7_: Contour corresponding to a 25% risk of transmission of influenza from one guinea pig to another over 7 days; 25-hD-25-hydroxyvitamin D; AC: Air conditioning; C: Centigrade; HEPA: High efficiency particle absorbance; HVAC: Heating ventilating and air conditioning; RH: Relative humidity; SARS: Severe acute respiratory syndrome; T: Temperature; URTI: Upper respiratory tract infection; UV: Ultraviolet light

## Competing interests

The authors declare that they have no competing interests.

## Authors' contributions

BB participated in the design of the study, collected data and contributed to writing. BPH conceived the study, collected data, analyzed results, and drafted the manuscript. All authors read and approved the final manuscript.

## Authors' information

BB is a PhD biochemist who has worked extensively with guided evolution systems and cellular pathway engineering. BPH is a PhD microbiologist working with assay systems and is also a computer scientist, developer of the Epiflex epidemic modeling software.

## Supplementary Material

Additional file 1 **Contagion contour estimation details**. Technical guide to equations and detail discussions for modelers and analysts. Click here for file

Additional file 2 Empirical 25% line equation in text format for use in Excel. Click here for file

Additional file 3 Maple workbook for 25% line equation. Click here for file

Additional file 4 Maple workbook for contagion probability equation. Click here for file

Additional file 5 Empirical contagion probability integral in text format for use in Excel. Click here for file
