# Supplementary material for: Aerosol influenza transmission risk contours: A study of humid tropics versus winter temperate zone
Source: Virol J. 2010 May 14;7:98. doi: 10.1186/1743-422X-7-98 (PMC2893155; doi:10.1186/1743-422X-7-98)
Supplement: Additional file 1 — Contagion contour estimation details. Technical guide to equations and detail discussions for modelers and analysts. [file 1743-422X-7-98-S1.PDF]

Brian P. Hanley§ and Birthe Borup

Contagion contour estimation and equation discussions

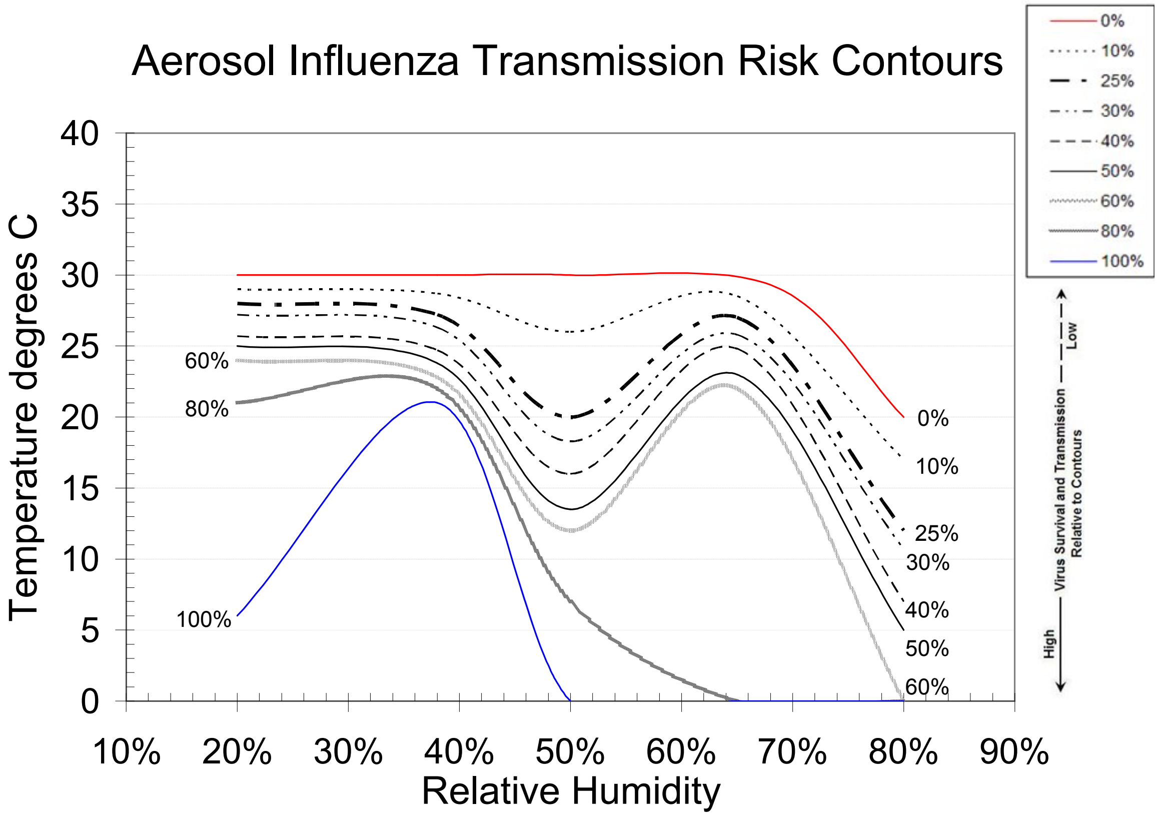

Figure 1 – Contagion contours
The 25%$_{G7}$ transmission contour is emphasized as a boundary for locations inside or outside of high risk contagion, with low risk above and increasing risk below.

Transmission risk contour map development method

Contours were developed by interpolation of Lowen et al.[1, 2] in combination with other sources. Figures extracted from these papers were set into a table and averaged to produce a transmission risk for each relative humidity level (RH). From these data a smoothed curve plot was generated using Excel for each temperature. For each of the three temperatures of Lowen (5°, 20° and 30° C), a relative humidity (RH) level and a transmission risk was determined and put into a second table, categorized by RH. Three linearly interpolated temperatures (10°, 15° and 25° C) were also entered into the second table for each RH level. The 65% RH at 20° C point was retained for reasons discussed below.

Similar tables were made for Schaffer et al.[3] and Harper et al.[4]. These worksheets are for a nominal temperature of 21° C, and correlate with the features from Lowen of a transmission trough around 50% RH. Schaffer's 60 minute viability data supports the rise in transmission for 65% RH at 20° C. Since survival of infectious particles directly drives ability to infect, we did not consider it valid to ignore this 65% RH feature of the Lowen data. We also consider it a conservative feature to include, and thus necessary.

A third table was filled in with data sets read from the second table for different transmission levels (0%, 10%, 25%, 30%, 40%, 50%, 60%, 80%, and 100%). For each transmission level, the temperature and RH for 20%, 35%, 50%, 65%, and 80% RH was read from the graph of the second table. After completing this third table, the 35% point was moved to 38% to remove artifacts when the curve was smoothed by Excel. The result is the contour map of figure 1.

§ Corresponding author email: brian.hanley@ieee.org

Brian P. Hanley§ and Birthe Borup

Statistical validity of the contour graph

As discussed by Lowen, the difference in transmissibility between 5 °C and 20 °C at 50% and 80% humidity is significant ($p < 0.05$) [2]. Thus, the 50% feature of Lowen is of sufficient statistical power as is the decline in transmissibility at 80%.

To further evaluate the Lowen data, we considered it in the context of Harper[4] and Schaffer[3] data on time course viability of influenza virions at differing temperature and humidity, because it is axiomatic that the longer virions can remain viable in aerosol, the more likely they are to cause infection by this route. Harper shows support for the decline with RH increasing toward 50%. Schaffer data [3] at one hour for 21 °C (see figure two of Schaffer et al.) shows a viability trough at 50% RH with rising viability at higher humidity followed by a decline. This pattern of Schaffer supports the Lowen 20 °C data for 50% RH. Additionally, this feature of Schaffer supports the 65% RH increase in transmission called out as statistically of insufficient power by Lowen. We asked ourselves how likely it was that these entirely different experiments with the same virus quasi-species would show such a correlation by chance and decided it was quite improbable. An additional argument for retention of the 65% RH increase in transmission is care to present conservative contagion contours where there is a question; thus we retained the feature showing a rise in contagion at 65% RH.

Consequently, although p values for Lowen alone are of insufficient power for the 65% RH rise in contagion, taking alternative data sources into account, we retained the 65% RH feature. We understand that the details of the type of contour map we present may change with larger datasets between 5 °C and 30 °C and we strongly encourage performance of larger experiments with multiple strains of influenza and other respiratory viruses. It would be highly desirable to have a larger dataset on the order of 30 animals or more at each temperature and RH setting. It would also be desirable to have a greater density of temperature and broader RH values in such studies, in addition to variance in airflow, room configurations, movement and proposed mitigations.

Empirical polynomial fit equations

Equation 1: $25\%_{G7}$ risk contour temperature

$$T = 9685.14588232279494 - 2.52084560221109656*10^{5}*h$$
$$+2.87816398667755630*10^{6}*h^{2} - 1.89131146527758204*10^{7}*h^{3}$$
$$+7.91750188318004161 10^{7} h^{4} - 2.20615774299511403 10^{8} h^{5}$$
$$+4.14577569357022464 10^{8} h^{6} - 5.19328079218272984 10^{8} h^{7}$$
$$+4.15621664851125360 10^{8} h^{8} - 1.92222450433581471 10^{8} h^{9}$$
$$+3.90844348204430044 10^{7} h^{10}$$
$$0.20 \le h \le 0.80$$

*Where **h** is RH and **T** is temperature at the $25\%_{G7}$ contour for the RH supplied. Use this equation to find the distance from the $25\%_{G7}$ contour of a (T, RH) pair. Do not exceed the limits of the equation for values of **h**.*

For the equation 1 Maple 10 polynomial fitted to the $25\%_{G7}$ transmission risk contour of figure 1, 13 coordinates were supplied where x was RH and y was temperature. Interpolations were read off the smoothed interpolation graph at 5% RH intervals to generate the 13 coordinates so that the polynomial equation could fit the contour more exactly since more points than the order of the fit equation are needed. Additional file 3, **Maple workbook for 25% line equation** is the Maple 10 file for equation 2.

The resulting polynomial equation was then translated into a text equation suitable to use in Excel. The Excel formatted version of the equation used can be seen in Additional file 2, **Empirical 25% line equation in text format for use in Excel**.

To use this equation, supply a value for RH, and it will return the temperature of the $25\%_{G7}$ transmission contour for that RH. The returned T value can be subtracted from a real absolute temperature to find the temperature distance of a field collected data point from the $25\%_{G7}$ contour. *Be careful not to exceed the limits of the equation.* RH values less than 20% and above 80% will return meaningless results.

§ Corresponding author email: brian.hanley@ieee.org

Equation 2: Contagion probability estimate by time expressed in days

$$p = \int_0^t 0.29406 + 0.01162416t - 0.04563274t^2 + 0.0098177t^3 - 0.00060869t^4 dt$$

$$0 \le t \le 8$$

$$p_c = C(T, RH) \cdot p$$

Where ***t*** *= time expressed in days,* ***p*** *is crude risk probability assuming 7 day contagion is 100%,* ***C(T,RH)*** *is the estimated risk for a field reading from the contour map and* $\mathbf{p_c}$ *= probability of contagion. Result* ***p*** *when multiplied by contagion contour percentage at a temperature and RH provides the time scaled risk of infection in a location (*$\mathbf{p_c}$*).*

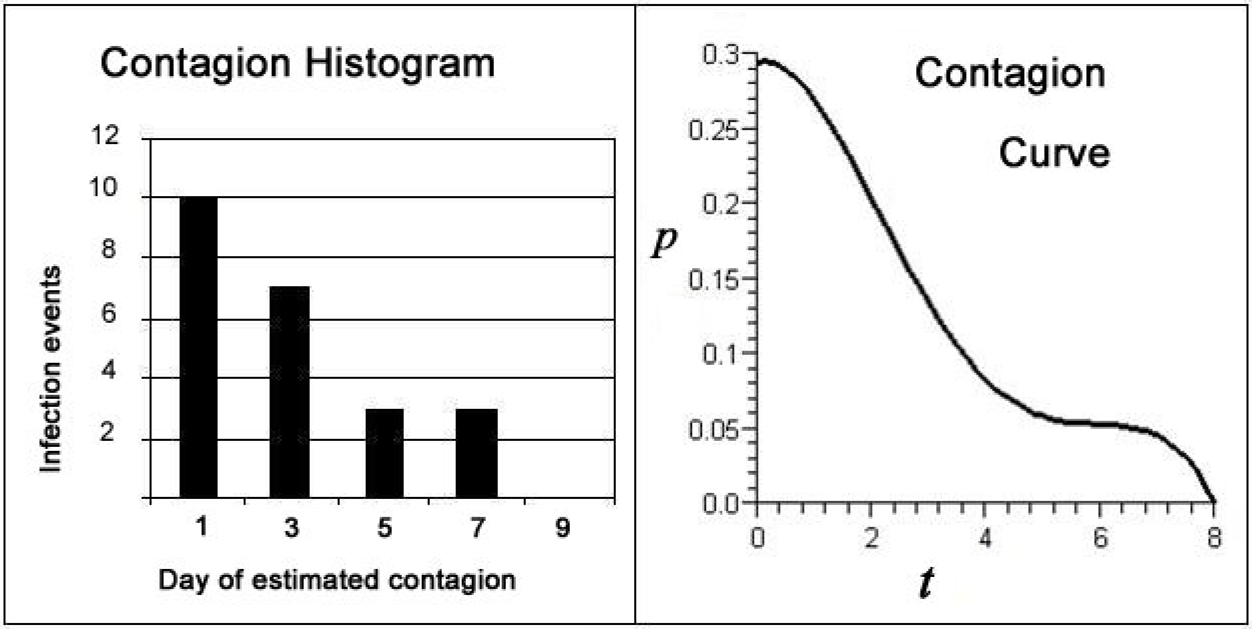

Figure 2 – Contagion histogram and curve plot. N = 23

Development of equation 2 started with a histogram of the day on which viral titers appeared in Lowen[2] as shown in figure 2. There are 23 secondary infections that occur in this distribution. Due to the expected length of viral illness in primary animals, secondary infection after the 8th to 9th days was not expected.

The contagion histogram data is represented to Maple as fractions of the total of 23 secondary infection events in the Contagion Histogram. Lowen introduced primary animals into environmental chambers with naïve animals 24 hours post inoculation. The incubation period in humans for influenza is estimated at 1-3 days post exposure. We used a period of 1.5 days post inoculation for significant virus shedding of primary inoculated guinea pigs[5], assuming a rise to maximum after 2 days. For the purposes of this equation, we assumed maximum shedding of virus as an initial condition, since this model is intended for generalized use rather than to follow a contagion curve for an individual over time. Thus, the values for the *t* axis were set from 0 to 8 to reflect the probable conditions at time of contagion rather than appearance of viral loads. This is also more convenient for integration. The 4th degree equation was preferred for its fit, and the integral iterated, multiplying each *p* axis input value by a fraction until the total area under the curve was equal to 1, as a value of 1 reflects a total probability of unity. Prior to iteration, the fractional *p* axis input values at the knee of the curve were slightly modified to produce a better fit. Normalization of the curve area to 1 was carried out to 3 decimal places.

§ Corresponding author email: brian.hanley@ieee.org

Additional file 4, **Maple workbook for empirical contagion probability equation** is the Maple 10 file for equation 2. The indefinite integral of equation 2 was then translated into an Excel format which is available in Additional file 5, **Empirical contagion probability integral in text format for use in Excel** for use by others.

To use equation 2, supply a time value expressed in days (which may be fractional). The result will be the probability of infection by aerosol in an environment where $100\%_{G7}$ infection is expected. Multiply the first result by the expected infection fraction (i.e. $25\%_{G7}$, $50\%_{G7}$, etc. or an interpolated value between risk contour lines) to get the probability of infection for a particular temperature and RH. *Be careful not to exceed the limits of the equation.* Supplying time values below 0 or above 8 will give meaningless results. Time values greater than 8 days should be estimated at zero.

Note that in the real world there are factors not fully accounted for by this estimation equation, and for modeling purposes, sometimes such factors should be accounted for. In addition to the earlier noted factor of the level of virus shedding (and hence contagiousness) of infected hosts over the time course of an illness, there are two other fundamental situations of exposure. In the first exposure situation, a naïve host enters an environment for a period of time where an infectious host has been resident for a sufficiently long period to reach an equilibrium level of aerosol virus load for the rate of exhalation versus rate of deactivation of virus. This situation is intended to be generally modeled by equation 2 above. In the second exposure situation, an infectious individual enters an environment of naïve hosts for a limited period of time, adding aerosol virus.

The example of the Alaskan Airlines aircraft with a 72% attack rate over 4.5 hours without ventilation[6] indicates the degree to which ventilation can impact aerosol transmission, since aerosol virus load per unit of air would be expected to vary based on rate of input, rate of deactivation, and degree of dilution by ventilation. A 2X to 10X multiplier on estimates from equation 2 is recommended where air exchange is poor, with 10X as a reasonable upper bound.

Since superspreaders are probably required to support an epidemic[7], and we believe that the first exposure situation is that of superspreaders, we decided that the proper conservative equation for general use is the one shown. Interested parties should contact the author for discussion of equations specific to the listed situations.

References

1. Lowen AC, Mubareka S, Steel J, Palese P: **Influenza virus transmission is dependent on relative humidity and temperature.** *PLoS Pathogens* 2007, **3:10:**1470-1476.
2. Lowen AC, Steel J, Mubareka S, Palese P: **High temperature (30°C) blocks aerosol but not contact transmission of influenza virus.** *Journal of Virology* 2008, **82:11:**5650-5652.
3. Schaffer FL, Soergel ME, Straube DC: **Survival of airborne influenza virus: effects of propagating host, relative humidity, and composition of spray fluids.** *Archives of Virology* 1976, **51:4:**263-273.
4. Harper GJ: **Airborne micro-organisms: survival tests with four viruses.** *The Journal of Hygiene* 1961, **59:**479-486.
5. Dettwiler HA, Hudson P, Woolpert OC: **The comparative susceptibility of fetal and postnatal guinea pigs to the virus of epidemic influenza.** *Journal of Experimental Medicine* 1940, **72:6:**623-634.
6. Moser MR, Bender TR, Margolis HS, Noble GR, Kendal AP, Ritter DG: **An outbreak of influenza aboard a commercial airliner** *American Journal of Epidemiology* 1979, **110:1:**1-6.
7. Lloyd-Smith JO, Schreiber SJ, Kopp PE, Getz WM: **Superspreading and the effect of individual variation on disease emergence.** *Nature* 2005, **438:**355-359.

§ Corresponding author email: brian.hanley@ieee.org
